# Supplementary material for: Out-of-distribution generalization for learning quantum dynamics
Source: Nat Commun. 2023 Jul 5;14:3751. doi: 10.1038/s41467-023-39381-w (PMC10322910; doi:10.1038/s41467-023-39381-w)
Supplement: Supplementary file 1 — Supplementary Information [file 41467_2023_39381_MOESM1_ESM.pdf]

Supplementary Information for "Out-of-Distribution Generalization for Learning Quantum Dynamics"

Supplementary Note 1. Preliminaries

Before beginning our main discussion, we introduce some notation that will be used throughout the Supplementary Material. In our discussion, we consider systems consisting of n qubits. Thus, we work with the complex Hilbert space $(\mathbb{C}^2)^{\otimes n}$ of dimension $d = 2^n$. For any d, $\mathcal{B}(\mathbb{C}^d)$ denotes the set of bounded linear operators on $\mathbb{C}^d$, which we implicitly identify with the set of $d \times d$ matrices by fixing a basis whenever convenient. Also, we denote by $\mathcal{U}(\mathbb{C}^d)$ the set of unitary operators on $\mathbb{C}^d$. The sets of operators $\mathcal{B}((\mathbb{C}^2)^{\otimes n})$ and $\mathcal{U}((\mathbb{C}^2)^{\otimes n})$ are defined analogously. Finally, we use standard bra-ket notation for pure quantum states.

We consider the task of learning an unknown n-qubit unitary $U \in \mathcal{U}((\mathbb{C}^2)^{\otimes n})$ from pairs of input and output states using a *quantum neural network (QNN)*. For our purposes, we think of a QNN as a (possibly variable-structure) k-local quantum circuit on n qubits that contains tunable gates. (Here, k is an n-independent constant). Mathematically, we describe such a QNN by a parameterized n-qubit unitary $V(\boldsymbol{\alpha})$ with classical parameters $\boldsymbol{\alpha}$, where the parameterization arises from the QNN structure. The parameter vector $\boldsymbol{\alpha}$ can consist of both continuous parameters (which indeed parameterize the trainable gates, e.g. acting as rotation angles) and discrete parameters (which encode freedom in the chosen quantum circuit structure, e.g. the number of trainable gates). The input to the quantum learning procedure is a training data set $\mathcal{D}_{\mathcal{Q}}(N)$ of the form

$$\mathcal{D}_{\mathcal{Q}}(N) = \{(|\Psi^{(j)}\rangle, |\Phi^{(j)}\rangle)\}_{j=1}^{N} \tag{1}$$

where the $|\Psi^{(j)}\rangle \in (\mathbb{C}^2)^{\otimes n}$ are pure n-qubit input states drawn i.i.d. from a probability distribution $\mathcal{Q}$ and $|\Phi^{(j)}\rangle = U|\Psi^{(j)}\rangle$ are the corresponding output states. The goal is to train the classical parameters $\boldsymbol{\alpha}$ in the QNN $V(\boldsymbol{\alpha})$ such that the QNN $V(\boldsymbol{\alpha}_{\mathrm{opt}})$ with the optimized parameters $\boldsymbol{\alpha}_{\mathrm{opt}}$ predicts the output states of U well on average when the input states are drawn from a testing probability distribution $\mathcal{P}$ over pure n-qubit states. That is, the optimized parameters $\boldsymbol{\alpha}_{\mathrm{opt}}$ should be such that

$$R_{\mathcal{P}}\left(U, V(\boldsymbol{\alpha}_{\mathrm{opt}})\right) \coloneqq \frac{1}{4}\mathbb{E}_{|\Psi\rangle\sim\mathcal{P}}\left[\left\|U|\Psi\rangle\langle\Psi|U^\dagger - V(\boldsymbol{\alpha}_{\mathrm{opt}})|\Psi\rangle\langle\Psi|V(\boldsymbol{\alpha}_{\mathrm{opt}})^\dagger\right\|_1^2\right] \tag{2}$$

$$= 1 - \mathbb{E}_{|\Psi\rangle\sim\mathcal{P}}\left[\left|\langle\Psi|U^\dagger V(\boldsymbol{\alpha}_{\mathrm{opt}})|\Psi\rangle\right|^2\right] \tag{3}$$

is small. A learner who does not know the testing distribution $\mathcal{P}$ and the target unitary U cannot evaluate the expected testing risk from Eq. (3). Instead, given a training data set as in Eq. (1), the learner may try to evaluate and optimize the training cost

$$C_{\mathcal{D}_{\mathcal{Q}}(N)}(U, V(\boldsymbol{\alpha})) \coloneqq \frac{1}{4N}\sum_{j=1}^{N}\left\|U|\Psi^{(j)}\rangle\langle\Psi^{(j)}|U^\dagger - V(\boldsymbol{\alpha})|\Psi^{(j)}\rangle\langle\Psi^{(j)}|V(\boldsymbol{\alpha})^\dagger\right\|_1^2 \tag{4}$$

$$= 1 - \frac{1}{N}\sum_{j=1}^{N}\left|\langle\Psi^{(j)}|U^\dagger V(\boldsymbol{\alpha})|\Psi^{(j)}\rangle\right|^2 . \tag{5}$$

Here, we rewrite the trace norm distance between two pure states in terms of their fidelity to obtain an expression for the training cost that can be evaluated on a quantum computer with a swap test [1, 2].

Optimizing the training cost from Eq. (5), however, is not automatically a promising avenue towards achieving a small expected testing risk from Eq. (3). Such a promise can only be fulfilled when a good performance on the available training data, i.e. a small value of $C_{\mathcal{D}_{\mathcal{Q}}(N)}(U, V(\boldsymbol{\alpha}_{\mathrm{opt}}))$, also leads to a good average performance on previously unseen data points, i.e. to a small value of $R_{\mathcal{P}}\left(U, V(\boldsymbol{\alpha}_{\mathrm{opt}})\right)$. This ability to *generalize* from training data to unseen data is of central importance to the viability of (quantum) machine learning. In particular, the generalization behavior often has a determining influence on the amount of training data that a (quantum) machine learning model requires.

For the case of $\mathcal{Q} = \mathcal{P}$, when training and testing data are drawn i.i.d. from the same distribution, such questions can be studied in the standard framework of *in-distribution generalization* (sometimes also known as *weak generalization*). Here, however, we focus on the case of $\mathcal{Q} \neq \mathcal{P}$, when the QNN is trained on a distribution different from the testing distribution. This scenario is variously known as *out-of-distribution generalization* or *strong generalization*. More precisely, we will consider training and testing states coming from (different) locally scrambled ensembles [3, 4].

Definition 1 (Locally scrambled ensembles – Restatement of Definition 1 from the main text)**.** An ensemble $\mathcal{U}$ of n-qubit unitaries is called *locally scrambled* if it is invariant under preprocessing by tensor products of arbitrary local unitaries. That is, if $U \sim \mathcal{U}$, then for any fixed $U_1, \ldots, U_n \in \mathcal{U}(\mathbb{C}^2)$ also $U(\bigotimes_{i=1}^n U_i) \sim \mathcal{U}$. Accordingly, an ensemble $\mathcal{S}$ of n-qubit quantum states is called locally scrambled if it is of the form $\mathcal{S} = \mathcal{U}|0\rangle^{\otimes n}$ for some locally scrambled ensemble $\mathcal{U}$ of n-qubit unitaries.

We use $\mathbb{U}_{\mathrm{LS}}$ to denote the class of all locally scrambled unitary ensembles and $\mathbb{S}_{\mathrm{LS}}$ to denote the class of all locally scrambled state ensembles. (Here, we suppress the number of qubits in the notation in favor of improved readability.)

As discussed in more detail below, examples of locally scrambled unitary ensembles include Haar-random n-qubit unitaries, tensor products of Haar-random single-qubit unitaries followed by some fixed n-qubit unitary, and unitaries implemented by random quantum circuits of some fixed depth, among others.

In fact, our results for locally scrambled ensembles below immediately extend to a slightly broader class of ensembles:

Definition 2. An ensemble $\mathcal{U}$ of n-qubit unitaries is called *locally scrambled up to (and including) complex second moments* if there exists an ensemble $\tilde{\mathcal{U}}$ of n-qubit unitaries such that the complex first and second moments of $\mathcal{U}$ agree with those of $\tilde{\mathcal{U}}$. That is, we have $\mathbb{E}_{U\sim\mathcal{U}}[U\rho U^\dagger] = \mathbb{E}_{\tilde{U}\sim\tilde{\mathcal{U}}}[\tilde{U}\rho\tilde{U}^\dagger]$ for all density matrices $\rho \in \mathcal{B}(\mathbb{C}^d)$ and $\mathbb{E}_{U\sim\mathcal{U}}[U^{\otimes 2}\rho(U^\dagger)^{\otimes 2}] = \mathbb{E}_{\tilde{U}\sim\tilde{\mathcal{U}}}[\tilde{U}^{\otimes 2}\rho(\tilde{U}^\dagger)^{\otimes 2}]$ for all density matrices $\rho \in \mathcal{B}((\mathbb{C}^d)^{\otimes 2})$. Accordingly, an ensemble $\mathcal{S}$ of n-qubit quantum states is called locally scrambled up to (and including) complex second moments if it is of the form $\mathcal{S} = \mathcal{U}|0\rangle^{\otimes n}$ for ensemble $\mathcal{U}$ of n-qubit unitaries that is locally scrambled up to (and including) complex second moments.

We use $\mathbb{U}_{\mathrm{LS}}^{(2)}$ to denote the class of all unitary ensembles that are locally scrambled up to (and including) complex second moments and $\mathbb{S}_{\mathrm{LS}}^{(2)}$ to denote the class of all state ensembles that are locally scrambled up to (and including) complex second moments. (Again, we suppress the number of qubits in the notation in favor of improved readability.)

To illustrate the above definitions here we list examples of distribution in $\mathbb{S}_{\mathrm{LS}}$ and $\mathbb{S}_{\mathrm{LS}}^{(2)}$ and explain why they are such distributions.

Example 1 (Products of Haar-random single-qubit states, $\mathcal{S}_{\mathrm{Haar}_1^{\otimes n}}$). Define $\mathcal{S}_{\mathrm{Haar}_1^{\otimes n}} := \mathcal{U}_{\mathrm{Haar}_1^{\otimes n}}|0\rangle^{\otimes n}$, where $\mathcal{U}_{\mathrm{Haar}_1^{\otimes n}}$ is the n-fold tensor product of the single-qubit Haar measure on $\mathcal{U}(\mathbb{C}^2)$. By the right-invariance of $\mathcal{U}_{\mathrm{Haar}_1}$ under multiplication with an arbitrary fixed single-qubit unitary we have that $\mathcal{U}_{\mathrm{Haar}_1^{\otimes n}} \in \mathbb{U}_{\mathrm{LS}}$ and hence $\mathcal{S}_{\mathrm{Haar}_1^{\otimes n}} \in \mathbb{S}_{\mathrm{LS}}$.

Example 2 (Products of random single-qubit stabilizer states, $\mathcal{S}_{\mathrm{Stab}_1^{\otimes n}}$). Consider the ensemble composed of tensor products of random single-qubit stabilizer states, i.e. $\mathcal{S}_{\mathrm{Stab}_1^{\otimes n}} := \mathrm{Uniform}(\{|0\rangle, |1\rangle, |+\rangle, |-\rangle, |y+\rangle, |y-\rangle\}^{\otimes n})$. This ensemble agrees with $\mathcal{S}_{\mathrm{Haar}_1^{\otimes n}}$ up to the second moment and hence is in $\mathbb{S}_{\mathrm{LS}}^{(2)}$.

Example 3 (Post-processed tensor products of Haar-random single-qubit states). Consider an ensemble of n-qubit unitaries of the form $U\mathcal{U}_{\mathrm{Haar}_1^{\otimes n}}$, where $\mathcal{U}_{\mathrm{Haar}_1^{\otimes n}}$ denotes the n-fold tensor product of the single-qubit Haar measure on $\mathcal{U}(\mathbb{C}^2)$ and $U \in \mathcal{U}((\mathbb{C}^2)^{\otimes n})$ is some fixed unitary. Then, by right-invariance of $\mathcal{U}_{\mathrm{Haar}_1}$ under multiplication with an arbitrary fixed single-qubit unitary, we have $U\mathcal{U}_{\mathrm{Haar}_1^{\otimes n}}(\bigotimes_{i=1}^n U_i) = U\mathcal{U}_{\mathrm{Haar}_1^{\otimes n}}$, for any fixed unitaries $U_1, \ldots, U_n \in \mathcal{U}(\mathbb{C}^2)$. Thus, $U\mathcal{U}_{\mathrm{Haar}_1^{\otimes n}}$ is a locally scrambled ensemble of n-qubit unitaries and $U\mathcal{U}_{\mathrm{Haar}_1^{\otimes n}}|0\rangle^{\otimes n} \in \mathbb{S}_{\mathrm{LS}}$.

Example 4 (Haar-random n-qubit states, $\mathcal{S}_{\mathrm{Haar}_n}$). Haar-random n-qubit states, i.e. $\mathcal{S}_{\mathrm{Haar}} := \mathcal{U}_{\mathrm{Haar}_n}|0\rangle^{\otimes n}$, form a locally scrambled ensemble because the Haar measure on $\mathcal{U}((\mathbb{C}^2)^{\otimes n})$ is (both left- and) right-invariant under multiplication with an arbitrary fixed n-qubit unitary. So, $\mathcal{U}_{\mathrm{Haar}_n} \in \mathbb{U}_{\mathrm{LS}}$ and $\mathcal{S}_{\mathrm{Haar}} \in \mathbb{S}_{\mathrm{LS}}$.

Example 5 (2-design on n-qubit states, $\mathcal{S}_{\mathrm{2design}}$). A unitary 2-design, by definition, agrees with the Haar distribution up to the second moment and hence $\mathcal{S}_{\mathrm{2design}} := \mathcal{U}_2|0\rangle^{\otimes n} \in \mathbb{S}_{\mathrm{LS}}^{(2)}$. Such distributions can be well approximated using a polynomial-depth hardware-efficient ansatz [5–7].

Example 6 (Products of Haar-random k-qubit states, $\mathcal{S}_{\mathrm{Haar}_k^{\otimes n/k}}$). For $n/k \in \mathbb{N}$ we can capture both $\mathcal{S}_{\mathrm{Haar}_n}$ and $\mathcal{S}_{\mathrm{Haar}_1^{\otimes n}}$ by more generally defining $\mathcal{S}_{\mathrm{Haar}_k^{\otimes n/k}} := \mathcal{U}_{\mathrm{Haar}_k^{\otimes n/k}}|0\rangle^{\otimes n}$, where $\mathcal{U}_{\mathrm{Haar}_1^{\otimes n/k}}$ is the n/k-fold tensor product of the k-qubit Haar measure on $\mathcal{U}((\mathbb{C}^2)^{\otimes k})$. Again, right-invariance of the Haar measure yields $\mathcal{S}_{\mathrm{Haar}_k^{\otimes n/k}} \in \mathbb{S}_{\mathrm{LS}}$.

Example 7 (Output states of random quantum circuits, $\mathcal{S}_{\mathrm{RandCirc}}^{\mathcal{A}_k}$). Let $\mathcal{A}_k$ be a k-local n-qubit quantum circuit architecture (in which every qubit is acted on non-trivially), with $k \leq n$. Let $\mathcal{U}_{\mathcal{A}_k}$ denote the ensemble of n-qubit unitaries obtained by drawing every k-qubit unitary in $\mathcal{A}_k$ at random from the k-qubit Haar measure. Then, by right-invariance of the k-qubit Haar measure, $\mathcal{U}_{\mathcal{A}_k} \in \mathbb{U}_{\mathrm{LS}}$. Accordingly, the ensemble $\mathcal{S}_{\mathrm{RandCirc}} := \mathcal{U}_{\mathcal{A}_k}|0\rangle^{\otimes n}$ of output states of a random quantum circuit with architecture $\mathcal{A}_k$ satisfies $\mathcal{S}_{\mathrm{RandCirc}}^{\mathcal{A}_k} \in \mathbb{S}_{\mathrm{LS}}$.

Supplementary Note 2. Analytical Results

1. Equivalence of Locally Scrambled Risks for Comparing Unitaries

We begin our analysis by comparing the testing risks obtained from Eq. (3) for different locally scrambled testing distributions. In this subsection, we show that all such testing risks are equivalent in the sense that they differ by at most a constant factor. We prove this equivalence by showing that all locally scrambled risks $R_{\mathcal{P}}(U,V)$ are tightly related to the Hilbert-Schmidt inner product between U and V. To formalize this discussion, we first introduce a cost arising naturally from that inner product:

Definition 3 (Hilbert-Schmidt test cost)**.** The *Hilbert-Schmidt test (HST) cost* between two n-qubit unitaries $U \in \mathcal{U}((\mathbb{C}^2)^{\otimes n})$ and $V \in \mathcal{U}((\mathbb{C}^2)^{\otimes n})$ is defined as

$$C_{\text{HST}}(U,V) \coloneqq 1 - \frac{1}{d^2}|\operatorname{Tr}[U^\dagger V]|^2 \,. \tag{6}$$

At this point, we note that, as shown in Refs. [8, 9], we can view the HST cost as an expected testing risk as in Eq. (3) via

$$C_{\text{HST}}(U,V) = \frac{d+1}{d} R_{\mathcal{S}_{\text{Haar}_n}}(U,V)\,, \tag{7}$$

where $d = 2^n$. (Here, the notation $R_{\mathcal{S}_{\text{Haar}_n}}(U,V)$ indicates an expected testing risk w.r.t. the Haar ensemble from Example 4.) In the main text, for conciseness of presentation, we have used the right hand-side of Eq. (7) instead of its left-hand side.

Our first result is an expression for the squared absolute value of the Hilbert-Schmidt inner product between two matrices – which in particular gives an expression for the HST cost between two unitaries – in terms of an average over n-qubit Paulis:

Lemma 1. *Let $n \in \mathbb{N}$ and write $d = 2^n$. Let $A, B \in \mathcal{B}(\mathbb{C}^d)$. Then,*

$$|\operatorname{Tr}[A^\dagger B]|^2 = \frac{1}{d} \sum_{P \in \{\mathbb{1},X,Y,Z\}^{\otimes n}} \operatorname{Tr}\left[PA^\dagger BPB^\dagger A\right] \,. \tag{8}$$

In particular, we can express the HST cost between two n-qubit unitaries $U \in \mathcal{U}((\mathbb{C}^2)^{\otimes n})$ and $V \in \mathcal{U}((\mathbb{C}^2)^{\otimes n})$ as

$$C_{\text{HST}}(U,V) = 1 - \frac{1}{d^3} \sum_{P \in \{\mathbb{1},X,Y,Z\}^{\otimes n}} \operatorname{Tr}\left[PU^\dagger VPV^\dagger U\right] \,. \tag{9}$$

Proof. Using the shorthand $C = A^\dagger B \in \mathcal{B}(\mathbb{C}^d)$, we have

$$\begin{aligned}
|\operatorname{Tr}[A^\dagger B]|^2 &= |\operatorname{Tr}[C]|^2 && (10)\\
&= \operatorname{Tr}[C]\overline{\operatorname{Tr}[C]} && (11)\\
&= \operatorname{Tr}[C]\operatorname{Tr}[C^\dagger] && (12)\\
&= \operatorname{Tr}[C \otimes C^\dagger] && (13)\\
&= \operatorname{Tr}[\text{SWAP}^2(C \otimes C^\dagger)] && (14)\\
&= \frac{1}{d} \sum_{P \in \{\mathbb{1},X,Y,Z\}^{\otimes n}} \operatorname{Tr}[\text{SWAP}(PC \otimes PC^\dagger)] && (15)\\
&= \frac{1}{d} \sum_{P \in \{\mathbb{1},X,Y,Z\}^{\otimes n}} \operatorname{Tr}[PCPC^\dagger]\,. && (16)
\end{aligned}$$

Here, the first line is due to our shorthand, the second line is $|z|^2 = z\overline{z}$ for $z \in \mathbb{C}$, the third line uses $\operatorname{Tr}[C^\dagger] = \overline{\operatorname{Tr}[C]}$, the fourth line uses $\operatorname{Tr}[D \otimes E] = \operatorname{Tr}[D] \otimes \operatorname{Tr}[E]$, the fifth line uses $\text{SWAP}^2 = \mathbb{1}$, the sixth line uses the basis expansion $\text{SWAP} = \frac{1}{d}\sum_{P \in \{\mathbb{1},X,Y,Z\}^{\otimes n}} P \otimes P$ of the swap operator in the Pauli string basis, and the last line uses $\operatorname{Tr}[\text{SWAP}(D \otimes E)] = \operatorname{Tr}[DE]$. This establishes Eq. (8). Now, plugging Eq. (8) into the definition of the HST cost gives Eq. (9). □

Next, we present a technical lemma which we later use to control the Pauli average in the expression for the HST cost between two unitaries:

Lemma 2. *Let $n \in \mathbb{N}$ and write $d = 2^n$. Let $W \in \mathcal{U}((\mathbb{C}^2)^{\otimes n})$, let $P \in \{\mathbb{1}, X, Y, Z\}^{\otimes n}$, and let $|s\rangle \in \{|0\rangle, |1\rangle, |+\rangle, |-\rangle, |y+\rangle, |y-\rangle\}^{\otimes n}$ be an eigenvector of P. Then,*

$$0 \leq 1 - \langle s|P|s\rangle \cdot \langle s|W^\dagger P W|s\rangle \leq 2\left(1 - |\langle s|W|s\rangle|^2\right) . \tag{17}$$

Proof. Let $|s_1\rangle = |s\rangle, |s_2\rangle, \ldots, |s_d\rangle$ be an orthonormal basis consisting of eigenvectors of P. Then, by plugging in the spectral decomposition $P = \sum_{i=1}^d \langle s_i|P|s_i\rangle \cdot |s_i\rangle\langle s_i|$, we get, using that the eigenvalues of P lie in $\{-1, 1\}$,

$$\langle s|P|s\rangle \cdot \langle s|W^\dagger P W|s\rangle = \sum_{i=1}^d \langle s|P|s\rangle \cdot \langle s_i|P|s_i\rangle \cdot \langle s|W^\dagger|s_i\rangle\langle s_i|W|s\rangle \tag{18}$$

$$= \langle s|P|s\rangle^2 \cdot \langle s|W^\dagger|s\rangle\langle s|W|s\rangle + \sum_{i=2}^d \langle s|P|s\rangle \cdot \langle s_i|P|s_i\rangle \cdot \langle s|W^\dagger|s_i\rangle\langle s_i|W|s\rangle \tag{19}$$

$$= \underbrace{|\langle s|W|s\rangle|^2}_{=:p_1} + \sum_{i=2}^d \underbrace{\langle s|P|s\rangle \cdot \langle s_i|P|s_i\rangle}_{\in\{-1,1\}} \cdot \underbrace{\langle s|W^\dagger|s_i\rangle\langle s_i|W|s\rangle}_{=:p_i} . \tag{20}$$

With this notation, we have $0 \leq p_i \leq 1$ for all $1 \leq i \leq d$, where the upper bound holds by Cauchy-Schwarz and unitarity of W, as well as $\sum_{i=1}^d p_i = \langle s|W^\dagger W|s\rangle = \langle s|s\rangle = 1$, by unitarity of W. Thus, $\{p_i\}_{i=1}^d$ is a probability vector of length d. Therefore, from Eq. (20), we conclude

$$\langle s|P|s\rangle \cdot \langle s|W^\dagger P W|s\rangle \in [p_1 - (1 - p_1), p_1 + (1 - p_1)] = [2p_1 - 1, 1] . \tag{21}$$

Accordingly, we obtain

$$1 - \langle s|P|s\rangle \cdot \langle s|W^\dagger P W|s\rangle \in [0, 1 - (2p_1 - 1)] = [0, 2(1 - p_1)] , \tag{22}$$

as claimed. □

We emphasize that this proof, in contrast to those of Lemma 1 (and also Lemma 3 below), explicitly uses the unitarity of the matrix W. This is also why we assume unitarity of U and V in Lemma 4 below, since we will again consider $W = U^\dagger V$.

Lemmas 1 and 2 allow us to prove the following upper bound on the HST cost in terms of an average over tensor products of Haar-random single-qubit states:

Corollary 1. *Let $n \in \mathbb{N}$ and write $d = 2^n$. Let $U, V, \tilde{U} \in \mathcal{U}((\mathbb{C}^2)^{\otimes n})$. Then,*

$$C_{\text{HST}}(U, V) \leq 2\left(1 - \mathbb{E}_{\bigotimes_{i=1}^n |\psi_i\rangle \sim \text{Haar}_1^{\otimes n}}\left[\left|\left(\bigotimes_{i=1}^n \langle\psi_i|\right) \tilde{U}^\dagger W \tilde{U} \left(\bigotimes_{i=1}^n |\psi_i\rangle\right)\right|^2\right]\right) , \tag{23}$$

where we again use the shorthand $W = U^\dagger V$.

Proof. We begin with the expression for the HST cost in terms of a Pauli average derived in Lemma 1. For any fixed $\tilde{U} \in \mathcal{U}((\mathbb{C}^2)^{\otimes n})$, the definition of $C_{\text{HST}}(U, V)$ and Lemma 1 imply:

$$C_{\text{HST}}(U, V) = C_{\text{HST}}(\tilde{U}^\dagger U \tilde{U}, \tilde{U}^\dagger V \tilde{U}) = 1 - \frac{1}{d^3} \sum_{P \in \{\mathbb{1}, X, Y, Z\}^{\otimes n}} \operatorname{Tr}\left[P \tilde{U}^\dagger W \tilde{U} P \tilde{U}^\dagger W^\dagger \tilde{U}\right] . \tag{24}$$

Now, we can consider a spectral decomposition for $P \in \{\mathbb{1}, X, Y, Z\}^{\otimes n}$, which – since we are dealing with Pauli strings – we can write as follows:

$$P = \sum_{|s\rangle \in \{|0\rangle, |1\rangle, |+\rangle, |-\rangle, |y+\rangle, |y-\rangle\}^{\otimes n} : \langle s|P|s\rangle \neq 0} \langle s|P|s\rangle \cdot |s\rangle\langle s| . \tag{25}$$

Plugging this spectral decomposition into Eq. (9) to evaluate the trace, we obtain

$$C_{\text{HST}}(U,V) = 1 - \frac{1}{d^3}\sum_{P\in\{\mathbb{1},X,Y,Z\}^{\otimes n}} \operatorname{Tr}\left[P\tilde{U}^\dagger W\tilde{U}P\tilde{U}^\dagger W^\dagger \tilde{U}\right] \tag{26}$$

$$= 1 - \frac{1}{d^2}\sum_{P\in\{\mathbb{1},X,Y,Z\}^{\otimes n}} \frac{1}{d}\sum_{|s\rangle\in\{|0\rangle,|1\rangle,|+\rangle,|-\rangle,|y+\rangle,|y-\rangle\}^{\otimes n}:\langle s|P|s\rangle\neq 0} \operatorname{Tr}\left[P\tilde{U}^\dagger W\tilde{U}\left(\langle s|P|s\rangle\cdot |s\rangle\langle s|\right)\tilde{U}^\dagger W^\dagger\tilde{U}\right] \tag{27}$$

$$= 1 - \frac{1}{d^2}\sum_{P\in\{\mathbb{1},X,Y,Z\}^{\otimes n}} \frac{1}{d}\sum_{|s\rangle\in\{|0\rangle,|1\rangle,|+\rangle,|-\rangle,|y+\rangle,|y-\rangle\}^{\otimes n}:\langle s|P|s\rangle\neq 0} \langle s|P|s\rangle\langle s|\tilde{U}^\dagger W^\dagger\tilde{U}P\tilde{U}^\dagger W\tilde{U}|s\rangle \tag{28}$$

$$= \mathbb{E}_{P\sim\{\mathbb{1},X,Y,Z\}^{\otimes n}}\mathbb{E}_{|s\rangle\sim\{|0\rangle,|1\rangle,|+\rangle,|-\rangle,|y+\rangle,|y-\rangle\}^{\otimes n}:\langle s|P|s\rangle\neq 0}\left[1-\langle s|P|s\rangle\cdot\langle s|\tilde{U}^\dagger W^\dagger\tilde{U}P\tilde{U}^\dagger W\tilde{U}|s\rangle\right] \tag{29}$$

$$= \mathbb{E}_{|s\rangle\sim\{|0\rangle,|1\rangle,|+\rangle,|-\rangle,|y+\rangle,|y-\rangle\}^{\otimes n}}\mathbb{E}_{P\sim\{\mathbb{1},X,Y,Z\}^{\otimes n}:\langle s|P|s\rangle\neq 0}\left[1-\langle s|P|s\rangle\cdot\langle s|\tilde{U}^\dagger W^\dagger\tilde{U}P\tilde{U}^\dagger W\tilde{U}|s\rangle\right]. \tag{30}$$

Here, we denote by $\mathbb{E}_{P\sim\{\mathbb{1},X,Y,Z\}^{\otimes n}}$ the expectation over uniformly random Pauli strings of length n, and $\mathbb{E}_{|s\rangle\sim\{|0\rangle,|1\rangle,|+\rangle,|-\rangle,|y+\rangle,|y-\rangle\}^{\otimes n}:\langle s|P|s\rangle\neq 0}$ denotes the expectation over uniformly random tensor products of single-qubit stabilizer states which have non-vanishing overlap with P. Equivalently, the latter is the expectation over uniformly random eigenvectors of P. Similarly, $\mathbb{E}_{|s\rangle\sim\{|0\rangle,|1\rangle,|+\rangle,|-\rangle,|y+\rangle,|y-\rangle\}^{\otimes n}}$ denotes the expectation over uniformly random tensor products of single-qubit stabilizer states, and $\mathbb{E}_{P\sim\{\mathbb{1},X,Y,Z\}^{\otimes n}:\langle s|P|s\rangle\neq 0}$ denotes the expectation over uniformly random Pauli strings of length n that have non-vanishing overlap with $|s\rangle$. Equivalently, the latter is the expectation over uniformly random Pauli strings that have $|s\rangle$ as an eigenvector. Note that the expectation values involved here are w.r.t. uniform distributions over finite sets, which in particular justifies the last step in the above computation (since this then becomes a mere reordering of a finite sum). Plugging in the upper bound of Lemma 2, applied for the unitary $\tilde{U}^\dagger W\tilde{U}$, we further obtain:

$$C_{\text{HST}}(U,V) \leq 2\mathbb{E}_{|s\rangle\sim\{|0\rangle,|1\rangle,|+\rangle,|-\rangle,|y+\rangle,|y-\rangle\}^{\otimes n}}\mathbb{E}_{P\sim\{\mathbb{1},X,Y,Z\}^{\otimes n}:\langle s|P|s\rangle\neq 0}\left[1-|\langle s|\tilde{U}^\dagger W\tilde{U}|s\rangle|^2\right] \tag{31}$$

$$= 2\left(1-\mathbb{E}_{|s\rangle\sim\{|0\rangle,|1\rangle,|+\rangle,|-\rangle,|y+\rangle,|y-\rangle\}^{\otimes n}}\left[|\langle s|\tilde{U}^\dagger W\tilde{U}|s\rangle|^2\right]\right) \tag{32}$$

$$= 2\left(1-\mathbb{E}_{\bigotimes_{i=1}^n|\psi_i\rangle\sim\text{Haar}_1^{\otimes n}}\left[\left|\left(\bigotimes_{i=1}^n\langle\psi_i|\right)\tilde{U}^\dagger W\tilde{U}\left(\bigotimes_{i=1}^n|\psi_i\rangle\right)\right|^2\right]\right), \tag{33}$$

where the last equality uses that single-qubit stabilizer states form a 2-design (compare, e.g., [10], or see [11–13] for a stronger statement). □

To facilitate the comparison between the HST cost and a locally scrambled risk, we next show how to rewrite a general locally scrambled risk:

Lemma 3. *Let $n\in\mathbb{N}$. Let $\mathcal{P}$ be a locally scrambled ensemble of n-qubit quantum states, with $\mathcal{U}_{\text{test}}$ the corresponding locally scrambled unitary ensemble. Then, for any n-qubit unitaries U and V, using the shorthand $W=U^\dagger V$,*

$$R_{\mathcal{P}}(U,V) = 1-\frac{1}{6^n}\sum_{A\subseteq\{1,\ldots,n\}}\mathbb{E}_{\tilde{U}\sim\mathcal{U}_{\text{test}}}\left[\left\|\operatorname{Tr}_{A^c}\left[\tilde{U}^\dagger W\tilde{U}\right]\right\|_F^2\right], \tag{34}$$

where Tr_{A^c} denotes partial trace over all systems with index not in the set A and $\|\cdot\|_F$ denotes the Frobenius norm (which is the norm induced by the Hilbert-Schmidt inner product).

Proof. Throughout the proof, we use the shorthand $W=U^\dagger V$. We begin by noticing that, since $\mathcal{U}$ is locally scrambled, for any fixed $U_1,\ldots,U_n\in\mathcal{U}(\mathbb{C}^2)$, we have:

$$R_{\mathcal{P}}(U,V) = 1-\mathbb{E}_{|\Psi\rangle\sim\mathcal{P}}\left[|\langle\Psi|W|\Psi\rangle|^2\right] \tag{35}$$

$$= 1-\mathbb{E}_{\tilde{U}\sim\mathcal{U}_{\text{test}}}\left[\left|\langle 0|^{\otimes n}\tilde{U}^\dagger W\tilde{U}|0\rangle^{\otimes n}\right|^2\right] \tag{36}$$

$$= 1-\mathbb{E}_{\tilde{U}\sim\mathcal{U}_{\text{test}}}\left[\left|\langle 0|^{\otimes n}\left(\bigotimes_{i=1}^n U_i^\dagger\right)\tilde{U}^\dagger W\tilde{U}\left(\bigotimes_{i=1}^n U_i\right)|0\rangle^{\otimes n}\right|^2\right]. \tag{37}$$

If we now take an expectation over tensor products of Haar-random single-qubit unitaries $\bigotimes_{i=1}^n U_i \sim \mathrm{Haar}_1^{\otimes n}$, this yields:

$$R_{\mathcal{P}}(U,V) = 1 - \mathbb{E}_{\bigotimes_{i=1}^n U_i \sim \mathrm{Haar}_1^{\otimes n}} \mathbb{E}_{\tilde{U}\sim\mathcal{U}_{\mathrm{test}}} \left[\left| \langle 0|^{\otimes n} \left(\bigotimes_{i=1}^n U_i^\dagger \right) \tilde{U}^\dagger W \tilde{U} \left(\bigotimes_{i=1}^n U_i \right) |0\rangle^{\otimes n} \right|^2 \right] \tag{38}$$

$$= 1 - \mathbb{E}_{\tilde{U}\sim\mathcal{U}_{\mathrm{test}}} \mathbb{E}_{\bigotimes_{i=1}^n U_i \sim \mathrm{Haar}_1^{\otimes n}} \left[\left| \langle 0|^{\otimes n} \left(\bigotimes_{i=1}^n U_i^\dagger \right) \tilde{U}^\dagger W \tilde{U} \left(\bigotimes_{i=1}^n U_i \right) |0\rangle^{\otimes n} \right|^2 \right] \tag{39}$$

$$= \mathbb{E}_{\tilde{U}\sim\mathcal{U}_{\mathrm{test}}} \left[1 - \mathbb{E}_{\bigotimes_{i=1}^n |\psi_i\rangle \sim \mathrm{Haar}_1^{\otimes n}} \left[\left| \left(\bigotimes_{i=1}^n \langle \psi_i| \right) \tilde{U}^\dagger W \tilde{U} \left(\bigotimes_{i=1}^n |\psi_i\rangle \right) \right|^2 \right] \right] \tag{40}$$

$$= \mathbb{E}_{\tilde{U}\sim\mathcal{U}_{\mathrm{test}}} \left[1 - \mathbb{E}_{\bigotimes_{i=1}^n |\psi_i\rangle \sim \mathrm{Haar}_1^{\otimes n}} \left[\mathrm{Tr} \left[\left(\bigotimes_{i=1}^n |\psi_i\rangle\langle\psi_i| \right) \tilde{U}^\dagger W \tilde{U} \left(\bigotimes_{i=1}^n |\psi_i\rangle\langle\psi_i| \right) \tilde{U}^\dagger W^\dagger \tilde{U} \right] \right] \right], \tag{41}$$

where used that we can exchange the order of the expectation values by Tonelli's theorem, since the integrand is non-negative, and then slightly abused notation by using $\mathrm{Haar}_1^{\otimes n}$ to also denote the probability distribution describing a tensor product of Haar-random single-qubit states.

Next, we recall the following Haar identity (see, e.g., Eq. (2.26) in [14]):

$$\mathbb{E}_{|\psi\rangle\sim\mathrm{Haar}_1} \left[|\psi\rangle\langle\psi|^{\otimes 2} \right] = \frac{\mathbb{1}\otimes\mathbb{1} + \mathrm{SWAP}}{6}, \tag{42}$$

where SWAP is the swap operator between two qubits and $\mathbb{1}$ denotes the identity matrix on a single qubit system. Using this identity and the trace equality

$$\mathrm{Tr}\left[\mathrm{SWAP}(A\otimes B)\right] = \mathrm{Tr}\left[AB\right], \tag{43}$$

we can rewrite, for any fixed $\tilde{U}\in\mathcal{U}((\mathbb{C}^2)^{\otimes n})$,

$$\mathbb{E}_{\bigotimes_{i=1}^n |\psi_i\rangle \sim \mathrm{Haar}_1^{\otimes n}} \left[\mathrm{Tr} \left[\left(\bigotimes_{i=1}^n |\psi_i\rangle\langle\psi_i| \right) \tilde{U}^\dagger W \tilde{U} \left(\bigotimes_{i=1}^n |\psi_i\rangle\langle\psi_i| \right) \tilde{U}^\dagger W^\dagger \tilde{U} \right] \right] \tag{44}$$

$$= \mathbb{E}_{\bigotimes_{i=1}^n |\psi_i\rangle \sim \mathrm{Haar}_1^{\otimes n}} \left[\mathrm{Tr} \left[\left(\bigotimes_{i=1}^n \mathrm{SWAP}_{i,i} \right) \left(\left(\bigotimes_{i=1}^n |\psi_i\rangle\langle\psi_i| \right) \tilde{U}^\dagger W \tilde{U} \right) \otimes \left(\left(\bigotimes_{i=1}^n |\psi_i\rangle\langle\psi_i| \right) \tilde{U}^\dagger W^\dagger \tilde{U} \right) \right] \right] \tag{45}$$

$$= \mathrm{Tr} \left[\bigotimes_{i=1}^n \left(\mathrm{SWAP}_{i,i} \frac{\mathbb{1}_i\otimes\mathbb{1}_i + \mathrm{SWAP}_{i,i}}{6} \right) (\tilde{U}^\dagger W \tilde{U} \otimes \tilde{U}^\dagger W^\dagger \tilde{U}) \right]. \tag{46}$$

Here, $\mathrm{SWAP}_{i,i}$ denotes the single-qubit swap operator that acts on the ith qubits of the first and second n-qubit tensor factors, respectively. Next, we use that the swap operator is its own inverse and the definition of the partial trace to continue the computation:

$$\mathrm{Tr} \left[\bigotimes_{i=1}^n \left(\mathrm{SWAP}_{i,i} \frac{\mathbb{1}_i\otimes\mathbb{1}_i + \mathrm{SWAP}_{i,i}}{6} \right) (\tilde{U}^\dagger W \tilde{U} \otimes \tilde{U}^\dagger W^\dagger \tilde{U}) \right] \tag{47}$$

$$= \mathrm{Tr} \left[\bigotimes_{i=1}^n \left(\frac{\mathbb{1}_i\otimes\mathbb{1}_i + \mathrm{SWAP}_{i,i}}{6} \right) (\tilde{U}^\dagger W \tilde{U} \otimes \tilde{U}^\dagger W^\dagger \tilde{U}) \right] \tag{48}$$

$$= \frac{1}{6^n} \sum_{A\subseteq\{1,\ldots,n\}} \mathrm{Tr} \left[\left(\left(\bigotimes_{i\notin A} (\mathbb{1}_i\otimes\mathbb{1}_i) \right) \otimes \left(\bigotimes_{i\in A} \mathrm{SWAP}_{i,i} \right) \right) (\tilde{U}^\dagger W \tilde{U} \otimes \tilde{U}^\dagger W^\dagger \tilde{U}) \right] \tag{49}$$

$$= \frac{1}{6^n} \sum_{A\subseteq\{1,\ldots,n\}} \mathrm{Tr} \left[\left(\bigotimes_{i\in A} \mathrm{SWAP}_{i,i} \right) \mathrm{Tr}_{A^c,A^c} \left[\tilde{U}^\dagger W \tilde{U} \otimes \tilde{U}^\dagger W^\dagger \tilde{U} \right] \right]. \tag{50}$$

In the last step, we have used Tr_{A^c,A^c} to denote the partial trace over the A^c-subsystems of both the first and the second n-qubit tensor factors. Observing that $\mathrm{Tr}_{A^c,A^c}\left[\tilde{U}^\dagger W \tilde{U} \otimes \tilde{U}^\dagger W^\dagger \tilde{U}\right] = \mathrm{Tr}_{A^c}[\tilde{U}^\dagger W \tilde{U}] \otimes \mathrm{Tr}_{A^c}[\tilde{U}^\dagger W^\dagger \tilde{U}]$ and

writing $\mathrm{SWAP}_{A,A} = \bigotimes_{i\in A} \mathrm{SWAP}_{i,i}$, we finish the computation and obtain

$$\frac{1}{6^n} \sum_{A\subseteq\{1,\dots,n\}} \mathrm{Tr}\left[\left(\bigotimes_{i\in A} \mathrm{SWAP}_{i,i}\right) \mathrm{Tr}_{A^c,A^c}\left[\tilde{U}^\dagger W \tilde{U} \otimes \tilde{U}^\dagger W^\dagger \tilde{U}\right]\right] \tag{51}$$

$$= \frac{1}{6^n} \sum_{A\subseteq\{1,\dots,n\}} \mathrm{Tr}\left[\mathrm{SWAP}_{A,A}(\mathrm{Tr}_{A^c}[\tilde{U}^\dagger W \tilde{U}] \otimes \mathrm{Tr}_{A^c}[\tilde{U}^\dagger W^\dagger \tilde{U}])\right] \tag{52}$$

$$= \frac{1}{6^n} \sum_{A\subseteq\{1,\dots,n\}} \mathrm{Tr}\left[\mathrm{Tr}_{A^c}\left[\tilde{U}^\dagger W \tilde{U}\right] \mathrm{Tr}_{A^c}\left[\tilde{U}^\dagger W^\dagger \tilde{U}\right]\right] \tag{53}$$

$$= \frac{1}{6^n} \sum_{A\subseteq\{1,\dots,n\}} \left\|\mathrm{Tr}_{A^c}\left[\tilde{U}^\dagger W \tilde{U}\right]\right\|_F^2 . \tag{54}$$

Plugging this observation into Eq. (41) finishes the proof. □

As an aside, note that the proof of Lemma 3 does not use the unitarity of U and V. That is, Lemma 3, just like Lemma 1, is valid for arbitrary $U, V \in \mathcal{B}(\mathbb{C}^d)$. However, Lemma 2, Corollary 1, and the results from here on make use of the unitarity assumption.

Now, we are ready to combine the tools developed so far to establish the main technical result of this subsection. We show that all locally scrambled risks are equivalent to the HST cost:

Lemma 4 (Restatement of Lemma 1 from the main text)**.** *Let $n \in \mathbb{N}$ and write $d = 2^n$. Let $\mathcal{P}$ be a locally scrambled ensemble of n-qubit quantum states, with $\mathcal{U}_{\text{test}}$ the corresponding locally scrambled unitary ensemble. Then, for any n-qubit unitaries $U \in \mathcal{U}((\mathbb{C}^2)^{\otimes n})$ and $V \in \mathcal{U}((\mathbb{C}^2)^{\otimes n})$,*

$$\frac{1}{2} C_{\mathrm{HST}}(U,V) \leq R_{\mathcal{P}}(U,V) \leq C_{\mathrm{HST}}(U,V) . \tag{55}$$

Proof. Throughout the proof, we use the shorthand $W = U^\dagger V$. We start with the proof of the first inequality, $\frac{1}{2} C_{\mathrm{HST}}(U,V) \leq R_{\mathcal{P}}(U,V)$. To this end, we apply Corollary 1 to see that, for any $\tilde{U} \in \mathcal{U}((\mathbb{C}^2)^{\otimes n})$,

$$C_{\mathrm{HST}}(U,V) \leq 2\left(1 - \mathbb{E}_{\bigotimes_{i=1}^n |\psi_i\rangle \sim \mathrm{Haar}_1^{\otimes n}}\left[\left|\left(\bigotimes_{i=1}^n \langle\psi_i|\right) \tilde{U}^\dagger W \tilde{U} \left(\bigotimes_{i=1}^n |\psi_i\rangle\right)\right|^2\right]\right) . \tag{56}$$

Taking an expectation over $\tilde{U} \sim \mathcal{U}_{\text{test}}$, we obtain:

$$C_{\mathrm{HST}}(U,V) = \mathbb{E}_{\tilde{U}\sim\mathcal{U}_{\text{test}}}\left[C_{\mathrm{HST}}(\tilde{U}^\dagger U \tilde{U}, \tilde{U}^\dagger V \tilde{U})\right] \tag{57}$$

$$\leq 2\mathbb{E}_{\tilde{U}\sim\mathcal{U}_{\text{test}}}\left[1 - \mathbb{E}_{\bigotimes_{i=1}^n |\psi_i\rangle \sim \mathrm{Haar}_1^{\otimes n}}\left[\left|\left(\bigotimes_{i=1}^n \langle\psi_i|\right) \tilde{U}^\dagger W \tilde{U} \left(\bigotimes_{i=1}^n |\psi_i\rangle\right)\right|^2\right]\right] \tag{58}$$

$$= 2R_{\mathcal{P}}(U,V) , \tag{59}$$

where the last equality uses that $\mathcal{U}$ is locally scrambled and was already derived previously in Eq. (40). This finishes the proof of the first inequality.

We now turn our attention to the second inequality, $R_{\mathcal{P}}(U,V) \leq C_{\mathrm{HST}}(U,V)$. To prove this inequality, we rely on the expression for $R_{\mathcal{P}}(U,V)$ derived in Lemma 3. So, let $A \subseteq \{1,\dots,n\}$ be a subset of cardinality $|A| = k$. Then, we

have, again for any fixed $\tilde{U} \in \mathcal{U}(\mathbb{C}^d)$:

$$\begin{aligned}
\left\| \mathrm{Tr}_{A^c}\left[\tilde{U}^\dagger W \tilde{U}\right]\right\|_F^2 &= \sum_{i=1}^{2^k} \left(s_i\left(\mathrm{Tr}_{A^c}\left[\tilde{U}^\dagger W \tilde{U}\right]\right)\right)^2 && (60)\\
&\geq \frac{1}{2^k}\left(\sum_{i=1}^{2^k} s_i\left(\mathrm{Tr}_{A^c}\left[\tilde{U}^\dagger W \tilde{U}\right]\right)\right)^2 && (61)\\
&= \frac{1}{2^k}\left\|\mathrm{Tr}_{A^c}\left[\tilde{U}^\dagger W \tilde{U}\right]\right\|_1^2 && (62)\\
&\geq \frac{1}{2^k}\left|\mathrm{Tr}\left[\mathrm{Tr}_{A^c}\left[\tilde{U}^\dagger W \tilde{U}\right]\right]\right|^2 && (63)\\
&= \frac{1}{2^k}\left|\mathrm{Tr}\left[\tilde{U}^\dagger W \tilde{U}\right]\right|^2 && (64)\\
&= \frac{1}{2^k}\left|\mathrm{Tr}\left[W\right]\right|^2 , && (65)
\end{aligned}$$

where the first line is the definition of the Frobenius norm (also known as Schatten 2-norm) as 2-norm of the vector of singular values, the second line uses Jensen's inequality, the third line uses the definition of the trace norm (also known as Schatten 1-norm) as the sum of singular values, the fourth line uses Hölder's inequality (for $p = 1$ and $q = \infty$, i.e. $\mathrm{Tr}[AB] \leq ||A||_1 ||B||_\infty$ with $B = \mathbb{1}$), the fifth line uses that the trace of a partial trace equals the trace of the original matrix, and the last line uses unitarity of $\tilde{U}$ and the basis-invariance of the trace. Taking an expectation over $\tilde{U} \sim \mathcal{U}_{\mathrm{test}}$ and combining the resulting inequality with Lemma 3, we get

$$\begin{aligned}
R_{\mathcal{P}}(U,V) &= 1 - \frac{1}{6^n}\sum_{A\subseteq\{1,\ldots,n\}} \mathbb{E}_{\tilde{U}\sim\mathcal{U}_{\mathrm{test}}}\left[\left\|\mathrm{Tr}_{A^c}\left[\tilde{U}^\dagger W \tilde{U}\right]\right\|_F^2\right] && (66)\\
&\leq 1 - \frac{1}{6^n}\sum_{k=0}^{n}\binom{n}{k}\cdot\frac{1}{2^k}\left|\mathrm{Tr}\left[W\right]\right|^2 && (67)\\
&= 1 - \frac{1}{4^n}\left|\mathrm{Tr}\left[W\right]\right|^2 && (68)\\
&= C_{\mathrm{HST}}(U,V)\,. && (69)
\end{aligned}$$

This is the second inequality that we set out to prove. □

As an immediate consequence of Lemma 4, since all locally scrambled risks are equivalent to the HST cost, we also see that all locally scrambled risks are equivalent to each other up to a constant multiplicative factor. That is, the following holds.

Theorem 1 (Equivalence of locally scrambled ensembles for comparing unitaries – Restatement of Theorem 1 from the main text)**.** *Let $\mathcal{P}$ and $\mathcal{Q}$ be two locally scrambled ensembles of n-qubit quantum states. Then, for any n-qubit unitaries U and V,*

$$\frac{1}{2}R_{\mathcal{Q}}(U,V) \leq R_{\mathcal{P}}(U,V) \leq 2R_{\mathcal{Q}}(U,V)\ . \tag{70}$$

Thus, for the purposes of comparing unitaries, all locally scrambled ensembles are in effect equivalent. In the next subsection, we combine this insight with known in-distribution generalization bounds for learning unitaries via QNNs to establish out-of-distribution generalization guarantees, if both the training and the testing distribution are locally scrambled. In particular, we will use Theorem 1 to show that, if we train on input states coming from a "simple" locally scrambled ensemble, such as random product states (Example 1), and have good in-distribution-generalization there, then we generalize to any other "more complicated" locally scrambled ensemble, such as fully Haar-random (and thus highly entangled) states or output states of random circuits (Examples 4 and 7).

Remark 1. By definition, the expected risk in Eq. (3) depends only on the complex second moment of the testing distribution $\mathcal{P}$. Therefore, we can directly extend Theorem 1 beyond locally scrambled ensembles, i.e. elements of $\mathbb{S}_{\mathrm{LS}}$, to ensembles whose complex second moments agree with those of some locally scrambled ensembles, i.e. to elements of $\mathbb{S}_{\mathrm{LS}}^{(2)}$. As a concrete example (discussed also in Example 2): Single-qubit stabilizer states form a 2-design and tensor products of Haar-random single-qubit states are locally scrambled. Thus, also the testing risk obtained by taking tensor products of random single-qubit stabilizer states for $\mathcal{P}$ in Eq. (3) is equivalent to any locally scrambled risk up to factors of 2.

2. Out-of-Distribution Generalization for QNNs Trained on Locally Scrambled States

In this subsection, we use our results of Subsection 2.1. to strengthen in-distribution generalization bounds for learning unitaries via QNNs to out-of-distribution generalization bounds for the same task, where we allow for arbitrary locally scrambled training and testing distributions. The following theorem, where we denote the in-distribution generalization error by $\mathrm{gen}_{\mathcal{Q},\mathcal{D}_{\mathcal{Q}}(N)}(\boldsymbol{\alpha}) = R_{\mathcal{Q}}(U, V(\boldsymbol{\alpha})) - C_{\mathcal{D}_{\mathcal{Q}}(N)}(U, V(\boldsymbol{\alpha}))$, serves as a general template for such a strengthening:

Corollary 2 (Out-of-distribution generalization from in-distribution generalization in unitary learning – Restatement of Corollary 1 from the main text)**.** *Let $n \in \mathbb{N}$ and write $d = 2^n$. Let $\mathcal{Q}$ and $\mathcal{P}$ be two locally scrambled ensembles of n-qubit quantum states. Let U be an unknown n-qubit unitary. Let $V(\boldsymbol{\alpha})$ be an n-qubit unitary QNN. For any parameter setting $\boldsymbol{\alpha}$, we have*

$$R_{\mathcal{P}}(U, V(\boldsymbol{\alpha})) \leq 2\left(C_{\mathcal{D}_{\mathcal{Q}}(N)}(U, V(\boldsymbol{\alpha})) + \mathrm{gen}_{\mathcal{Q},\mathcal{D}_{\mathcal{Q}}(N)}(\boldsymbol{\alpha})\right). \tag{71}$$

Proof. As both $\mathcal{Q}$ and $\mathcal{P}$ are locally scrambled ensembles of n-qubit quantum states, Theorem 1 yields

$$R_{\mathcal{P}}(U, V(\boldsymbol{\alpha})) \leq 2R_{\mathcal{Q}}(U, V(\boldsymbol{\alpha})). \tag{72}$$

After rewriting

$$R_{\mathcal{Q}}(U, V(\boldsymbol{\alpha})) = C_{\mathcal{D}_{\mathcal{Q}}(N)}(U, V(\boldsymbol{\alpha})) + \left(R_{\mathcal{Q}}(U, V(\boldsymbol{\alpha})) - C_{\mathcal{D}_{\mathcal{Q}}(N)}(U, V(\boldsymbol{\alpha}))\right), \tag{73}$$

this gives the statement of the theorem. □

Corollary 2 has the following implication for out-of-distribution generalization after training: When training the QNN $V(\boldsymbol{\alpha})$ with the cost $C_{\mathcal{D}_{\mathcal{Q}}(N)}$ using training data $\mathcal{D}_{\mathcal{Q}}(N)$, the out-of-distribution testing risk $R_{\mathcal{P}}(U, V(\boldsymbol{\alpha}_{\mathrm{opt}}))$ of the parameter setting $\boldsymbol{\alpha}_{\mathrm{opt}}$ after training is controlled in terms of the training cost and the in-distribution generalization error. Here, the only assumption on the training and testing distributions is that they are both locally scrambled.

To demonstrate the usefulness of Corollary 2, we next show the concrete form it takes when combined with the QNN generalization guarantees of [15]:

Corollary 3 (Locally scrambled out-of-distribution generalization for QNNs - Restatement of Corollary 2 from the main text)**.** *Let $n \in \mathbb{N}$ and write $d = 2^n$. Let $\delta \in (0,1)$. Let $\mathcal{Q}$ and $\mathcal{P}$ be two locally scrambled ensembles of n-qubit quantum states. Let U be an unknown n-qubit unitary. Let $V(\boldsymbol{\alpha})$ be an n-qubit unitary QNN with T parameterized local gates. When trained with the cost $C_{\mathcal{D}_{\mathcal{Q}}(N)}$ using training data $\mathcal{D}_{\mathcal{Q}}(N)$, the out-of-distribution testing risk w.r.t. $\mathcal{P}$ of the parameter setting $\boldsymbol{\alpha}_{\mathrm{opt}}$ after training satisfies*

$$R_{\mathcal{P}}(U, V(\boldsymbol{\alpha}_{\mathrm{opt}})) \leq 2C_{\mathcal{D}_{\mathcal{Q}}(N)}(U, V(\boldsymbol{\alpha}_{\mathrm{opt}})) + \mathcal{O}\left(\sqrt{\frac{T\log(T)}{N}} + \sqrt{\frac{\log(1/\delta)}{N}}\right), \tag{74}$$

with probability $\geq 1-\delta$ over the choice of training data of size N according to $\mathcal{Q}$.

Proof. According to [15, Theorem 11], the in-distribution generalization error in this setting is bounded as

$$R_{\mathcal{Q}}(U, V(\boldsymbol{\alpha})) - C_{\mathcal{D}_{\mathcal{Q}}(N)}(U, V(\boldsymbol{\alpha})) \leq \mathcal{O}\left(\sqrt{\frac{T\log(T)}{N}} + \sqrt{\frac{\log(1/\delta)}{N}}\right). \tag{75}$$

Plugging this in-distribution generalization error bound into Corollary 2 yields the claim. □

Corollary 3 has the following implication for training data requirements: To ensure that, with high probability, the expected risk does not exceed twice the training cost by more than a specified accuracy ε, it suffices to have training data of size $\sim T\log(T)/\varepsilon^2$. This sufficient training data size scales only slightly superlinearly in the number of trainable gates in the QNN.

For the special case of the locally scrambled training ensemble being tensor products of Haar-random states, with corresponding product state training data given as

$$\mathcal{D}_{\mathrm{Haar}_1^{\otimes n}}(N) = \{(|\Psi^{(j)}_{\mathrm{Haar}_1^{\otimes n}}\rangle, |\Phi^{(j)}_{\mathrm{Haar}_1^{\otimes n}}\rangle)\}_{j=1}^N = \left\{\left(\bigotimes_{i=1}^n |\psi_i^{(j)}\rangle, U\left(\bigotimes_{i=1}^n |\psi_i^{(j)}\rangle\right)\right)\right\}_{j=1}^N, \tag{76}$$

where the $|\psi_i^{(j)}\rangle$ are independent Haar-random single-qubit states and U is the unknown target unitary, Corollary 3 becomes:

Corollary 4 (Out-of-distribution generalization for QNNs trained on random product states)**.** *Let $n \in \mathbb{N}$ and write $d = 2^n$. Let $\delta \in (0,1)$. Let $\mathcal{P}$ be a locally scrambled ensemble of n-qubit quantum states. Let U be an unknown n-qubit unitary. Let $V(\boldsymbol{\alpha})$ be an n-qubit unitary QNN with T parameterized local gates. When trained with the cost $C_{\mathcal{D}_{\mathrm{Haar}_1^{\otimes n}}(N)}$ using training data $\mathcal{D}_{\mathrm{Haar}_1^{\otimes n}}(N)$, the out-of-distribution risk w.r.t. $\mathcal{P}$ of the parameter setting $\boldsymbol{\alpha}_{\mathrm{opt}}$ after training satisfies*

$$R_{\mathcal{P}}(U, V(\boldsymbol{\alpha}_{\mathrm{opt}})) \leq 2C_{\mathcal{D}_{\mathrm{Haar}_1^{\otimes n}}(N)}(U, V(\boldsymbol{\alpha}_{\mathrm{opt}})) + \mathcal{O}\left(\sqrt{\frac{T\log(T)}{N}} + \sqrt{\frac{\log(1/\delta)}{N}}\right), \tag{77}$$

with probability $\geq 1-\delta$ over the choice of training data of size N according to $\mathcal{S}_{\mathrm{Haar}_1^{\otimes n}}$.

Notice that, following Remark 1, we can simplify the training data even further and still achieve the same performance. Namely, if we train on tensor products of random stabilizer states (instead of on tensor products of random product states), then exactly the same out-of-distribution generalization bound as in Corollary 4 holds.

Remark 2. We can extend our results for out-of-distribution generalization when training on tensor products of Haar-random states to local variants of our risks and costs. Such local costs are essential to avoid cost function dependent barren plateaus [16] when training a shallow QNN, thereby facilitating optimization. As a concrete example, when taking $\mathcal{S}_{\mathrm{Haar}_1^{\otimes n}}$ from Example 1 as testing ensemble, we can consider the local expected testing risk

$$R^L_{\mathcal{S}_{\mathrm{Haar}_1^{\otimes n}}}(U, V(\boldsymbol{\alpha})) = 1 - \mathbb{E}_{|\Psi_{\mathrm{P}}\rangle = \bigotimes_{i=1}^n |\psi_i\rangle \sim \mathcal{S}_{\mathrm{Haar}_1^{\otimes n}}}\left[\frac{1}{n}\sum_{i=1}^n \mathrm{Tr}\left[U|\Psi_{\mathrm{P}}^{(j)}\rangle\langle\Psi_{\mathrm{P}}^{(j)}|U^\dagger V(\boldsymbol{\alpha})\left(|\psi_i^{(j)}\rangle\langle\psi_i^{(j)}| \otimes \mathbb{1}_{\bar{i}}\right)V(\boldsymbol{\alpha})^\dagger\right]\right] \tag{78}$$

and, for a training data set as in Eq. (76), the local training cost

$$C^L_{\mathcal{D}_{\mathrm{Haar}_1^{\otimes n}}(N)}(U, V(\boldsymbol{\alpha})) = 1 - \frac{1}{nN}\sum_{j=1}^N\sum_{i=1}^n \mathrm{Tr}\left[U|\Psi_P^{(j)}\rangle\langle\Psi_P^{(j)}|U^\dagger V(\boldsymbol{\alpha})\left(|\psi_i^{(j)}\rangle\langle\psi_i^{(j)}| \otimes \mathbb{1}_{\bar{i}}\right)V(\boldsymbol{\alpha})^\dagger\right]. \tag{79}$$

Clearly, analogous local variants of expected risk and training cost can be defined whenever the respective ensemble has a tensor product structure. Among the examples presented in the main text, both $\mathcal{S}_{\mathrm{Haar}_1^{\otimes n}}$ from Example 1 and $\mathcal{S}_{\mathrm{Stab}_1^{\otimes n}}$ from Example 2 have that form. However, if the training data is highly entangled constructing such local costs in this manner is not possible. Thus, this is another important consequence of our proof that training on product state training data enjoys out-of-distribution generalization.

According to [8, Appendix C], we know that

$$R^L_{\mathcal{S}_{\mathrm{Haar}_1^{\otimes n}}}(U, V(\boldsymbol{\alpha})) \leq R_{\mathcal{S}_{\mathrm{Haar}_1^{\otimes n}}}(U, V(\boldsymbol{\alpha})) \leq n \cdot R^L_{\mathcal{S}_{\mathrm{Haar}_1^{\otimes n}}}(U, V(\boldsymbol{\alpha})). \tag{80}$$

We can combine this with Theorem 1 to obtain: If $\mathcal{P}$ is any locally scrambled ensemble of n-qubit quantum states, then for any n-qubit unitaries U and V,

$$\frac{1}{2}R^L_{\mathcal{S}_{\mathrm{Haar}_1^{\otimes n}}}(U, V(\boldsymbol{\alpha})) \leq R_{\mathcal{P}}(U, V(\boldsymbol{\alpha})) \leq 2nR^L_{\mathcal{S}_{\mathrm{Haar}_1^{\otimes n}}}(U, V(\boldsymbol{\alpha})). \tag{81}$$

With this observation, we can obtain Corollary 3 from the main text as a version of Corollary 4 when training on products of Haar-random single-qubit states, but now with the local cost from Eq. (79), simply replacing $C_{\mathcal{D}_{\mathrm{Haar}_1^{\otimes n}}(N)}(U, V(\boldsymbol{\alpha}_{\mathrm{opt}}))$ by $nC^L_{\mathcal{D}_{\mathrm{Haar}_1^{\otimes n}}(N)}(U, V(\boldsymbol{\alpha}_{\mathrm{opt}}))$ and $\mathcal{O}\left(\sqrt{\frac{T\log(T)}{N}} + \sqrt{\frac{\log(1/\delta)}{N}}\right)$ by $\mathcal{O}\left(n\sqrt{\frac{T\log(T)}{N}} + \sqrt{\frac{\log(1/\delta)}{N}}\right)$.

3. Remarks on the Role of Linearity

As outlined in the discussion between Corollaries 2 and 3 from the main text, linearity is important in enabling our out-of-distribution generalization. Intuitively, as long as the training states span the space on which one wishes to learn the action of the target unitary, it ought to be possible to train on those states and by linearity extrapolate to the entire space. However, this line of argument alone is insufficient to explain out-of-distribution generalization. The

random ensembles of states also have to be "well-behaved" to ensure good generalization from a manageable number of training states.

One way of highlighting this subtlety is to note that even an exponential number of computational basis states cannot be used to learn an unknown unitary using a cost formulated in terms of the 1-norm distance between the guess output and true output (or equivalently the fidelity between the guess and true outputs). Namely, computational basis states do not allow to learn relative phases. This can be illustrated by the following concrete example: Suppose the unknown unitary is the single-qubit unitary $U = e^{-i\varphi Z}$ for some $\varphi \in [0, 2\pi)$. That is, we consider

$$U = e^{-i\varphi} \begin{pmatrix} 1 & 0 \\ 0 & e^{2i\varphi} \end{pmatrix} . \tag{82}$$

The action of U on the computational basis states is thus given by $U|0\rangle = e^{-i\varphi}|0\rangle$ and $U|1\rangle = e^{i\varphi}|1\rangle$, all the relevant information lies in the relative phase between the two output states. As our notions of risk are (as is physically reasonable) independent of global phases in the output states, the unitary $V = \mathbb{1}_2$ achieves a perfect training cost on the training data set $\mathcal{D} = \{(|0\rangle, U|0\rangle), (|1\rangle, U|1\rangle)\}$, namely

$$\begin{aligned} C_{\mathcal{D}}(U,V) &= \frac{1}{8}\left(\left\|U|0\rangle\langle 0|U^\dagger - V|0\rangle\langle 0|V^\dagger\right\|_1^2 + \left\|U|1\rangle\langle 1|U^\dagger - V|1\rangle\langle 1|V^\dagger\right\|_1^2\right) && (83)\\ &= \frac{1}{8}\left(\left\||0\rangle\langle 0| - |0\rangle\langle 0|\right\|_1^2 + \left\||1\rangle\langle 1| - |1\rangle\langle 1|\right\|_1^2\right) && (84)\\ &= 0\,, && (85)\end{aligned}$$

and therefore, since the training data set in this case consists of exactly the two single-qubit computational basis states, a perfect expected testing risk over randomly drawn computational basis states, namely

$$\begin{aligned} R_{\mathcal{S}_{\mathrm{CompBasis}}}(U,V) &= \frac{1}{4}\mathbb{E}_{|\Psi\rangle\sim\mathcal{S}_{\mathrm{CompBasis}}}\left[\left\|U|\Psi\rangle\langle\Psi|U^\dagger - V|\Psi\rangle\langle\Psi|V^\dagger\right\|_1^2\right] && (86)\\ &= \frac{1}{8}\left(\left\|U|0\rangle\langle 0|U^\dagger - V|0\rangle\langle 0|V^\dagger\right\|_1^2 + \left\|U|1\rangle\langle 1|U^\dagger - V|1\rangle\langle 1|V^\dagger\right\|_1^2\right) && (87)\\ &= C_{\mathcal{D}}(U,V) && (88)\\ &= 0\,. && (89)\end{aligned}$$

However, V clearly fails to capture the relative phase between $U|0\rangle$ and $U|1\rangle$. In particular, if we consider the testing risk over uniformly random states in the X-basis, we see that

$$\begin{aligned} R_{\mathcal{S}_{X-\mathrm{Basis}}}(U,V) &= \frac{1}{4}\mathbb{E}_{|\Psi\rangle\sim\mathcal{S}_{X-\mathrm{Basis}}}\left[\left\|U|\Psi\rangle\langle\Psi|U^\dagger - V|\Psi\rangle\langle\Psi|V^\dagger\right\|_1^2\right] && (90)\\ &= \frac{1}{8}\left(\left\|U|+\rangle\langle +|U^\dagger - V|+\rangle\langle +|V^\dagger\right\|_1^2 + \left\|U|-\rangle\langle -|U^\dagger - V|-\rangle\langle -|V^\dagger\right\|_1^2\right) && (91)\\ &= \frac{1}{8}\left(\left\|U|+\rangle\langle +|U^\dagger - |+\rangle\langle +|\right\|_1^2 + \left\|U|-\rangle\langle -|U^\dagger - |-\rangle\langle -|\right\|_1^2\right) && (92)\\ &= \frac{1}{8}\left(\left\|\frac{1}{2}\left((e^{2i\varphi}-1)|1\rangle\langle 0| + (e^{-2i\varphi}-1)|0\rangle\langle 1|\right)\right\|_1^2 + \left\|\frac{1}{2}\left((1-e^{2i\varphi})|1\rangle\langle 0| + (1-e^{-2i\varphi})|0\rangle\langle 1|\right)\right\|_1^2\right) && (93)\\ &= \frac{1}{16}\left\|\begin{pmatrix} 0 & e^{-2i\varphi}-1 \\ e^{2i\varphi}-1 & 0\end{pmatrix}\right\|_1^2 && (94)\\ &= \frac{1-\cos(2\varphi)}{2} && (95)\\ &= \sin^2(\varphi)\,, && (96)\end{aligned}$$

which is strictly bigger than zero whenever φ is not an integer multiple of π. Also, using Eq. (7), we obtain

$$R_{\mathcal{S}_{\mathrm{Haar}}}(U,V) = \frac{2}{3}\left(1 - \frac{1}{4}|\mathrm{tr}\,[U^\dagger V]|^2\right) = \frac{2}{3}\sin^2(\varphi)\,, \tag{97}$$

which is strictly bigger than zero whenever φ is not an integer multiple of π. Thus, despite perfect training error, perfect in-distribution generalization error, and perfect in-distribution testing error, the out-of-distribution generalization and

testing errors can be non-zero. This single-qubit example shows that no analogue of Theorem 1 from the main text can hold without additional assumptions on shared properties between the training and testing ensembles (such as both being locally scrambled). In particular, a good training and testing performance on randomly drawn computational basis states does not imply a good testing performance over (for example) random X-basis states or Haar-random states. While this counterexample to out-of-distribution generalization from training on computational basis states is specific to our (physically motivated) choice of cost function, the above argument still emphasizes that linearity alone does not trivially imply out-of-distribution generalization.

It is further worth stressing that an argument based on linearity places no guarantees on how many training states are required/sufficient for convergence. The argument that 'as long as the training states span the space on which you wish to learn the action of the target unitary on, it ought to be possible to train on those states and by linearity extrapolate to the entire space' crucially only applies if you train on an exponentially large training ensemble. In general, how many states are required/sufficient to ensure good generalization will depend on the types of states in the training ensemble. In our work, we combine Theorem Theorem 1 from the main text with the recent in-distribution generalization bounds of [15] and thereby show that the worst-case training data requirements for random product states cannot be significantly worse than those for fully random states.

Supplementary Note 3. Additional Numerical Results

1. Numerical Test of Lemma 1 From the Main Text

We numerically probe the validity of Lemma 1 from the main text for the NISQ friendly scenario $\mathcal{Q} = \mathcal{S}_{\mathrm{Haar}_1^{\otimes n}}$, i.e. the set of Haar-random product states. In this case, the relation between the average Haar-random product state cost and the general n-qubit Haar-random state cost according to Lemma 1 from the main text is

$$R_{\mathcal{S}_{\mathrm{Haar}_1^{\otimes n}}}(U, V(\boldsymbol{\alpha})) \leq \frac{d+1}{d} R_{\mathcal{S}_{\mathrm{Haar}_n}}(U, V(\boldsymbol{\alpha})) \leq 2 R_{\mathcal{S}_{\mathrm{Haar}_1^{\otimes n}}}(U, V(\boldsymbol{\alpha})), \tag{98}$$

where $\boldsymbol{\alpha}$ contains all parameters that define the QNN. To make contact with NISQ applications, we will probe the validity of Ineq. (98) by sampling $W = V^\dagger U(\boldsymbol{\alpha})$ from random low-depth quantum circuits. Given W, we can evaluate $\frac{d+1}{d} R_{\mathcal{S}_{\mathrm{Haar}_n}}(U, V(\boldsymbol{\alpha}))$ from Eq. 7 and $R_{\mathcal{S}_{\mathrm{Haar}_1^{\otimes n}}}(U, V(\boldsymbol{\alpha}))$ from Lemma 3 with straightforward matrix operations. To ensure that we can quickly sample from a large range of cost values without the need for optimization, we implement an ansatz of the form

$$W(\boldsymbol{\alpha} = r \cdot \boldsymbol{\theta}) = \prod_{k=1}^{l} \left(\prod_{i=1}^{T} G_{ik}(r \cdot \boldsymbol{\theta_{ik}}) \right). \tag{99}$$

Here, each G_{ik} is an arbitrary 2-qubit gate, and the inner product over i represents a hardware-efficient tiling of these 2-qubit gates. That is, we apply $\lfloor n/2 \rfloor$ 2-qubit gates from even qubits to odd qubits (i.e. between $(0,1), (2,3), \mathrm{etc}\ldots$) in parallel and then $\lfloor (n-1)/2 \rfloor$ 2-qubit gates from odd to even (i.e. between $(1,2), (3,4), \mathrm{etc}\ldots$) in parallel. The outer product just means we are applying l layers. So far, we have just described a familiar hardware-efficient tiling of arbitrary 2-qubit gates used in many variational quantum algorithms [16–18], but there is one crucial difference between our implementation and the standard one. Rather than use the minimal 15 single-qubit gate and 3 CNOT gate decomposition of G [19, 20] (aka the KAK decomposition [21]), we use a slightly larger 21 single-qubit gate and 4 CNOT gate decomposition. Though our choice has more parameters than necessary, it is defined so that $G(\mathbf{0}) = I$, which is not true for the KAK decomposition. This has the desirable property that $W(\boldsymbol{\alpha} = \mathbf{0}) = I$. Of course, all risks/costs comparing U and V are defined so that when $W = V^\dagger U = I$, they vanish (i.e. $R = 0$ and $C = 0$ for any sensible risk R and cost C). By writing $\boldsymbol{\alpha} = r\boldsymbol{\theta}$ we emphasize the point that regardless of the choice of $\boldsymbol{\theta}$, setting $r = 0$ samples the point $(0, 0)$ where both risks vanish.

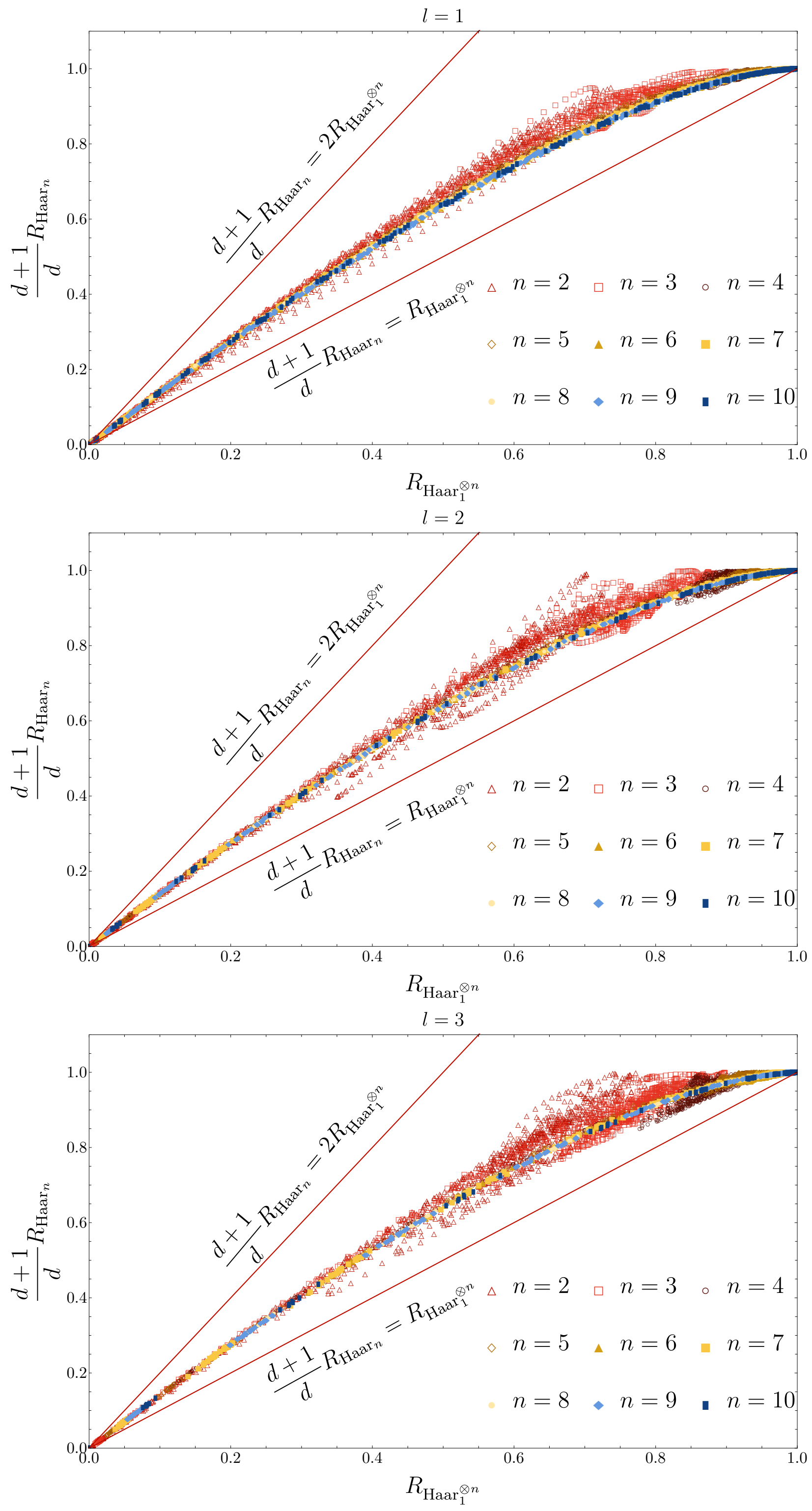

Supplementary Figure 1. We provide numerical verification of Lemma 1 from the main text for $\mathcal{Q} = \mathcal{S}_{\mathrm{Haar}_1^{\otimes n}}$, i.e. the ensemble of tensor products of Haar-random single-qubit states. Here, we consider the ansatz from Eq. (99) for one layer ($l = 1$, top panel), two layers ($l = 2$, middle panel), and three layers ($l = 3$, bottom panel). In this case, the claim is that Ineq. (98) should be satisfied for any circuit parameters $\boldsymbol{\alpha}$. Graphically, a sampled point satisfies the claimed Ineq. (98) if it lies between the two reference lines, which as expected is true for all generated points. Hence, learning the action of a unitary on random product states is sufficient to generalize to the entire Hilbert space in the very practical setting of low depth variational quantum circuits as shown here. It is also interesting to note that the upper-bound is empirically not very tight in these generic variational quantum circuits. In fact, the scaling of the locally trained cost to the global cost is empirically linear after sufficient training.

By defining the ansatz in this way, we can easily sample a large range of cost values. In particular, for fixed l and n, we randomly sample $(\boldsymbol{\theta_{ik}})_p \sim \mathcal{N}(0, 2\pi)\ \forall i, k, p$, generating a random initial parameter vector $\boldsymbol{\theta}^{(0)}$ for the entire ansatz from which we then compute the starting risks, $(R_{\mathcal{S}_{\mathrm{Haar}_1^{\otimes n}}}(U, V(\boldsymbol{\theta}^{(0)})), \frac{d+1}{d} R_{\mathcal{S}_{\mathrm{Haar}_n}}(U, V(\boldsymbol{\theta}^{(0)})))$. Note that this is the same initialization procedure for many variational quantum algorithms (VQAs) [16–18], and for a deep enough circuit with a random choice of $\boldsymbol{\theta}^{(0)}$, any risk or cost will be approximately maximized (i.e. $R \approx 1$ and $C \approx 1$). If we were truly running a VQA to learn $U(\boldsymbol{\theta})$ given a known unitary V, we would need to iteratively estimate $R_{\mathcal{S}_{\mathrm{Haar}_1^{\otimes n}}}$ on a quantum computer and update our best guess for $\boldsymbol{\theta}^*$ classically. Instead, we exploit the form of our toy ansatz directly: we simply re-scale each angle by r, i.e. $\boldsymbol{\theta}^{(0)} \to r\boldsymbol{\theta}^{(0)}$ for different values of r. By construction, the risk will be minimized when $r = 0$. Thus, by sampling values of $r \in [0, 1]$, we can explore the empirical cost relationship $(R_{\mathcal{S}_{\mathrm{Haar}_1^{\otimes n}}}(U, V(r \cdot \boldsymbol{\theta}^{(0)})), \frac{d+1}{d} R_{\mathcal{S}_{\mathrm{Haar}_n}}(U, V(r \cdot \boldsymbol{\theta}^{(0)})))$ between the two extremes. In Fig. 1, we show this empirical relationship for $n = 2, \ldots, 10$ qubits with ansatz depths $l = 1, 2, 3$ for 20 random initialization vectors $\boldsymbol{\theta}^{(i)}$ and 100 values r for each random sample. For all points sampled, Ineq. (98) is satisfied as expected. In fact, the upper-bound is often pretty loose, and it appears that a tighter relationship might be true even for large values of $R_{\mathcal{S}_{\mathrm{Haar}_1^{\otimes n}}}(U, V(\boldsymbol{\alpha}))$.

2. Out-of-Distribution Generalization for Learning Fast Scramblers

Task and setup: Here, we consider the task of learning a so-called fast scrambler [22] into an ansatz $V(\boldsymbol{\alpha})$ of a similar form. An n-qubit fast scrambler unitary U composed of t time steps is defined as follows:

$$U = \prod_{j=1}^{t} U_j^{\mathrm{I}} U_j^{\mathrm{II}} \ , \tag{100}$$

where U_j^{I} is a product of independent Haar-random single-qubit unitaries, $U_j^{\mathrm{I}} = \prod_{k=1}^{n} u_{j,k}$, and U_j^{II} is given by

$$U_j^{\mathrm{II}} = e^{-i\frac{g}{2\sqrt{n}}\sum_{k<\ell} Z_k Z_\ell} \ , \tag{101}$$

where g is a real parameter.

The ansatz $V(\boldsymbol{\alpha})$ for learning the scrambler U has the same structure as U with fixed single-qubit gates replaced by parametrized ones. That is, the ansatz $V(\boldsymbol{\alpha})$ takes the form

$$V(\boldsymbol{\alpha}) = \prod_{j=1}^{t} V_j^{\mathrm{I}}(\boldsymbol{\alpha}_j) U_j^{\mathrm{II}} \ , \tag{102}$$

where $V^{\mathrm{I}}(\boldsymbol{\alpha}_j) = \prod_{k=1}^{n} v_{j,k}(\boldsymbol{\alpha}_{j,k})$, with parametrized one qubit gates $v_{j,k}(\boldsymbol{\alpha}_{j,k})$. Here, we view t as the number of time steps. This is a parameter that controls the difficulty of the optimization problem, since the entanglement introduced by U quickly grows with t. The parameter g in Eq. (101) controls how quickly the learning difficulty grows with t. We work with $g = 1$, but consider several values of t.

The learning is performed as described in the main text. That is, we first build a training set and then optimize a corresponding cost function. First, we generate a training set of size N of the form $\mathcal{D}_{\mathcal{Q}}(N) = \{|\psi_j\rangle, U|\psi_j\rangle\}_{j=1}^{N}$, where input states $|\psi_j\rangle$ are random product states. Second, we optimize the parameters $\boldsymbol{\alpha}$ according to the cost function $C_{\mathcal{D}_{\mathcal{Q}}(N)}(\boldsymbol{\alpha})$ introduced in Eq. (3) of the main text. Optimized parameters $\boldsymbol{\alpha}_{\mathrm{opt}}$ are found by (approximately) solving the optimization problem:

$$\boldsymbol{\alpha}_{\mathrm{opt}} = \mathrm{argmin}_{\boldsymbol{\alpha}}\, C_{\mathcal{D}_{\mathcal{Q}}(N)}(\boldsymbol{\alpha}) \ . \tag{103}$$

We measure the learning quality with the (out-of-distribution) risk $R_{\mathcal{S}_{\mathrm{Haar}_n}}(\boldsymbol{\alpha})$, see Eq. (3) from the main text and also Eq. (7). We are interested in generalization error $R_{\mathcal{S}_{\mathrm{Haar}_n}}(\boldsymbol{\alpha}_{\mathrm{opt}}) - C_{\mathcal{D}_{\mathcal{Q}}(N)}(\boldsymbol{\alpha}_{\mathrm{opt}})$ as a function of various parameters in the learning problem.

Results: We learn an 8-qubit fast-scrambler unitary U with $t = 3, \ldots, 10$ and training data set sizes $N = 1, \ldots, 15$. The learning is performed by repeating the optimization in Eq. (103) 1000 times. Each optimization learns a different randomly generated U, works with different randomly drawn training set, and starts with different random initial parameters $\boldsymbol{\alpha}_0$. The optimization is performed with a variant of the gradient descent method in which the single-qubit unitaries $v_{j,k}(\boldsymbol{\beta})$ are spanned by three rotation angles, $v_{j,k}(\boldsymbol{\beta}) = e^{-iZ\beta_1} e^{-iX\beta_2} e^{-iZ\beta_3}$.

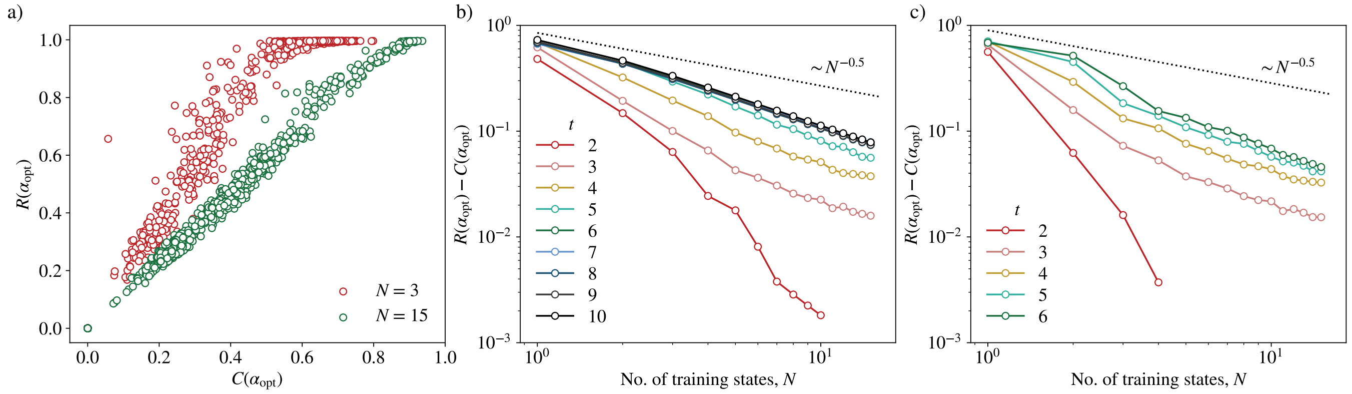

Supplementary Figure 2. **Learning an 8-qubit fast scrambler.** The individual panels extract scaling properties from the optimization data and show that our theoretical bounds apply, but are outperformed in practical learning tasks even for highly non-trivial, entangling unitaries. a) Testing risk $R_{\mathcal{S}_{\mathrm{Haar}_n}}(\boldsymbol{\alpha}_{\mathrm{opt}})$ as a function of the training cost $C_{\mathcal{D}_{\mathcal{Q}}(N)}(\boldsymbol{\alpha}_{\mathrm{opt}})$ calculated for 1000 independently obtained values of $\boldsymbol{\alpha}_{\mathrm{opt}}$. b) Average generalization error $R_{\mathcal{S}_{\mathrm{Haar}_n}}(\boldsymbol{\alpha}_{\mathrm{opt}}) - C_{\mathcal{D}_{\mathcal{Q}}(N)}(\boldsymbol{\alpha}_{\mathrm{opt}})$ over all 1000 optimizations as a function of N for several values of t. c) Average generalization error $R_{\mathcal{S}_{\mathrm{Haar}_n}}(\boldsymbol{\alpha}_{\mathrm{opt}}) - C_{\mathcal{D}_{\mathcal{Q}}(N)}(\boldsymbol{\alpha}_{\mathrm{opt}})$ over successful optimization runs as a function of N for several values of t. See text for an in-depth analysis.

Figure 2 summarizes our results. Panel (a) shows the testing risk $R_{\mathcal{S}_{\mathrm{Haar}_n}}(\boldsymbol{\alpha}_{\mathrm{opt}})$ as a function of the training cost $C_{\mathcal{D}_{\mathcal{Q}}(N)}(\boldsymbol{\alpha}_{\mathrm{opt}})$ calculated for 1000 independently obtained values of $\boldsymbol{\alpha}_{\mathrm{opt}}$. The data was obtained for $t = 5$. Blue (red) dots represent optimization performed with training data size $N = 3$ ($N = 15$). We observe that small training data size of $N = 3$ may lead to a situation in which optimization has already reached appreciable training cost values ($C_{\mathcal{D}_{\mathcal{Q}}(N)}(\boldsymbol{\alpha}_{\mathrm{opt}}) \approx 0.5$) but the testing risk is still at its maximal value ($R_{\mathcal{S}_{\mathrm{Haar}_n}}(\boldsymbol{\alpha}_{\mathrm{opt}}) \approx 1$). This generalization issue is resolved by adding more points to the training data set. Indeed, when training on $N = 15$ data points, the obtained data does not display a concentration around $R_{\mathcal{S}_{\mathrm{Haar}_n}}(\boldsymbol{\alpha}_{\mathrm{opt}}) \approx 1$. We also observe that larger data sets result in an increased likelihood of almost perfect learning (that is, an optimization that achieves $C_{\mathcal{D}_{\mathcal{Q}}(N)}(\boldsymbol{\alpha}_{\mathrm{opt}}) \simeq R_{\mathcal{S}_{\mathrm{Haar}_n}}(\boldsymbol{\alpha}_{\mathrm{opt}}) \simeq 0$). Only 7% of the optimization runs with $N = 3$ achieved almost perfect learning while 12.5% of the optimization runs with $N = 15$ reached that goal. That plot also shows that larger training set leads to better generalization: achieving a given cost value of $C_{\mathcal{D}_{\mathcal{Q}}(N)}(\boldsymbol{\alpha}_{\mathrm{opt}})$ with a bigger training set results in smaller risk $R_{\mathcal{S}_{\mathrm{Haar}_n}}(\boldsymbol{\alpha}_{\mathrm{opt}})$. We observe this behavior for every optimization that we have performed.

Panel (b) corroborates those findings further. It shows the generalization error $R_{\mathcal{S}_{\mathrm{Haar}_n}}(\boldsymbol{\alpha}_{\mathrm{opt}}) - C_{\mathcal{D}_{\mathcal{Q}}(N)}(\boldsymbol{\alpha}_{\mathrm{opt}})$, averaged over all 1000 optimizations, as a function of N, the training data size, for several values of t. We see that the average generalization error obtained for the values $t = 6, \ldots, 10$ is almost identical. The reason for this behavior is likely that for these values of t and for the training data sizes N used in our experiment, only very few optimization runs managed to lower the cost function enough to achieve a risk $R_{\mathcal{S}_{\mathrm{Haar}_n}}(\boldsymbol{\alpha}_{\mathrm{opt}})$ smaller than its maximal value. This optimization issue might be dealt with by more refined minimization techniques. While the results in this setup (large t and insufficiently large N) are not useful from a learning point of view, our theoretical generalization bounds still hold. As the data suggests, the scaling is better than the worst case scenario covered by the theoretical analysis.

Panel (c) avoids interpretational complications caused by optimization issues and averages only those minimization runs that achieved $C_{\mathcal{D}_{\mathcal{Q}}(N)}(\boldsymbol{\alpha}_{\mathrm{opt}}) < 0.5$. We see a scaling behavior similar to what we observed when taking the entire data into account. Panels (b) and (c) show that the generalization error decreases faster than theoretical upper bound, which scales as $\sim N^{-1/2}$ with the training data size N and is shown by the black solid line. As the learning difficulty (measured by t) increases, the rate at which generalization error decreases with N seems to slowly approach the theoretical bound.

[1] H. Buhrman, R. Cleve, J. Watrous, and R. De Wolf, Quantum fingerprinting, Physical Review Letters **87**, 167902 (2001).
[2] D. Gottesman and I. Chuang, Quantum digital signatures, arXiv preprint quant-ph/0105032 (2001).
[3] W.-T. Kuo, A. Akhtar, D. P. Arovas, and Y.-Z. You, Markovian entanglement dynamics under locally scrambled quantum evolution, Physical Review B **101**, 224202 (2020).
[4] H.-Y. Hu, S. Choi, and Y.-Z. You, Classical shadow tomography with locally scrambled quantum dynamics, Physical Review Research **5**, 023027 (2023).
[5] F. G. Brandao, A. W. Harrow, and M. Horodecki, Local random quantum circuits are approximate polynomial-designs, Communications in Mathematical Physics **346**, 397 (2016).
[6] A. Harrow and S. Mehraban, Approximate unitary t-designs by short random quantum circuits using nearest-neighbor and long-range gates, arXiv preprint arXiv:1809.06957 (2018).
[7] J. Haferkamp, Random quantum circuits are approximate unitary t-designs in depth $O\left(nt^{5+o(1)}\right)$, Quantum **6**, 795 (2022).
[8] S. Khatri, R. LaRose, A. Poremba, L. Cincio, A. T. Sornborger, and P. J. Coles, Quantum-assisted quantum compiling, Quantum **3**, 140 (2019).
[9] M. A. Nielsen, A simple formula for the average gate fidelity of a quantum dynamical operation, Physics Letters A **303**, 249 (2002).
[10] D. Gross, K. Audenaert, and J. Eisert, Evenly distributed unitaries: On the structure of unitary designs, Journal of mathematical physics **48**, 052104 (2007).
[11] R. Kueng and D. Gross, Qubit stabilizer states are complex projective 3-designs, arXiv preprint arXiv:1510.02767 (2015).
[12] Z. Webb, The clifford group forms a unitary 3-design, Quantum Information and Computation **16**, 1379 (2016).
[13] H. Zhu, Multiqubit clifford groups are unitary 3-designs, Physical Review A **96**, 062336 (2017).
[14] D. A. Roberts and B. Yoshida, Chaos and complexity by design, Journal of High Energy Physics **2017**, 121 (2017).
[15] M. C. Caro, H.-Y. Huang, M. Cerezo, K. Sharma, A. Sornborger, L. Cincio, and P. J. Coles, Generalization in quantum machine learning from few training data, Nature Communications **13**, 4919 (2022).
[16] M. Cerezo, A. Sone, T. Volkoff, L. Cincio, and P. J. Coles, Cost function dependent barren plateaus in shallow parametrized quantum circuits, Nature Communications **12**, 1 (2021).
[17] C. Cirstoiu, Z. Holmes, J. Iosue, L. Cincio, P. J. Coles, and A. Sornborger, Variational fast forwarding for quantum simulation beyond the coherence time, npj Quantum Information **6**, 1 (2020).
[18] J. Gibbs, K. Gili, Z. Holmes, B. Commeau, A. Arrasmith, L. Cincio, P. J. Coles, and A. Sornborger, Long-time simulations for fixed input states on quantum hardware, npj Quantum Information **8**, 135 (2022).
[19] F. Vatan and C. Williams, Optimal quantum circuits for general two-qubit gates, Physical Review A **69**, 032315 (2004).
[20] V. V. Shende, I. L. Markov, and S. S. Bullock, Minimal universal two-qubit controlled-not-based circuits, Physical Review A **69**, 062321 (2004).
[21] R. R. Tucci, An Introduction to Cartan's KAK Decomposition for QC Programmers, arXiv:quant-ph/0507171 (2005).
[22] R. Belyansky, P. Bienias, Y. A. Kharkov, A. V. Gorshkov, and B. Swingle, Minimal model for fast scrambling, Physical Review Letters **125**, 130601 (2020).
